# Case Report on Rapunzel syndrome: a large gastric trichobezoar extending to the proximal jejunum in a young adult female

**DOI:** 10.3389/fmed.2025.1504822

**Published:** 2025-03-19

**Authors:** Ksenija Mijović, Dragan Vasin, Sanela Hasanagić, Jelica Vukmirović, Tijana Tomić, Vasko Tošić, Aleksandar Pavlović, Dragan Mašulović, Aleksandra Đurić Stefanović

**Affiliations:** ^1^Center for Radiology, University Clinical Center of Serbia, Belgrade, Serbia; ^2^School of Medicine, University of Belgrade, Belgrade, Serbia; ^3^Emergency Surgery Clinic, University Clinical Center of Serbia, Belgrade, Serbia; ^4^Department of Digestive Radiology (First Surgery University Clinic), University Clinical Center of Serbia, Belgrade, Serbia

**Keywords:** trichobezoar, Rapunzel syndrome, gastric outlet obstruction, trichophagia, gastrotomy, case report

## Abstract

A trichobezoar is an intraluminal mass of hair growing continuously with additional ingestion, while Rapunzel syndrome refers to a giant gastric trichobezoar that extends from the stomach into the small intestine. We present a case of Rapunzel syndrome as an uncommon cause of upper gastrointestinal (GI) symptoms in a young adult woman who denied trichotillomania and trichophagia. Preoperative radiological assessment was pivotal in planning a preferable therapeutic approach. The patient underwent laparotomy and prepyloric gastrotomy, resulting in satisfactory postoperative outcomes. Trichobezoars are extremely rare and predominantly affect young women with underlying psychiatric conditions. Although uncommon, they cause severe upper gastrointestinal symptoms and may even lead to various complications. This case report helps in understanding the presentation of gastric trichobezoar and Rapunzel syndrome, including their range of symptoms, radiological appearance, and associated findings, to make an accurate diagnosis and guide an appropriate treatment approach.

## Introduction

A bezoar is an intraluminal mass formed by the accumulation of undigested material in the gastrointestinal (GI) tract (1). It consists of undigestible material, most commonly vegetable fibers (phytobezoar). However, other materials such as medication (pharmacobezoar) and undigested milk (lactobezoar) or foreign materials such as hair (trichobezoar) can also lead to the formation of a bezoar. This bezoar grows with the continuous supply of fiber-rich foods containing cellulose, which mix with protein and mucus (1–3). If a trichobezoar has a tail that extends through the pylorus into the small bowel or even the colon, the condition is referred to as Rapunzel syndrome (4).

Gastrointestinal (GI) trichobezoars are extremely rare, consisting of approximately 6% of all bezoars (5).

The majority of patients presenting with this condition are young women who experience trichotillomania (compulsive hair pulling) and trichophagia (hair ingestion) (6). These behaviors are generally associated with underlying psychiatric disorders such as depression, anxiety disorders, obsessive-compulsive disorder, child abuse, intellectual disability, and bereavement (7). However, this is not always the case, and the condition may also affect healthy individuals (5, 8).

We present a case of Rapunzel syndrome—a trichobezoar extending through the pylorus into the duodenum and jejunum—as an uncommon cause of intermittent epigastric pain, nausea, and vomiting in a young woman without any known underlying psychiatric conditions who also denied trichotillomania and trichophagia.

We believe that this case report will contribute to expanding medical knowledge of this rare gastrointestinal condition and improve the understanding of its epidemiology and clinical presentation.

## Case description

A 20-year-old woman presented to the emergency room (ER) with intermittent localized epigastric pain and vomiting that had started 1 month earlier. She reported having passed her last stool earlier that day, which was well-formed and unremarkable.

She underwent an appendectomy 5 years ago and had no other significant medical history.

Clinical examination revealed palpable epigastric pain with a large mass in the epigastrium, extending beneath the left rib cage toward the umbilicus, with no signs of peritoneal irritation.

The laboratory tests indicated leukocytosis, with a white blood cell count of 15.9×10^9^/l, and neutrophilia, while the patient’s C-reactive protein levels were within the reference range (1.8 mg/L). The patient also exhibited signs of microcytic anemia (with a mean corpuscular volume of 81.9 fL, mean corpuscular hemoglobin of 27.30 pg, and hemoglobin level of 130 g/L).

An abdominal ultrasound was performed, demonstrating suboptimal examination conditions due to artifacts arising from a distended stomach containing a large amount of air, interposed by an intraluminal foreign body. The “small bowel feces sign” was noted, suggesting delayed bowel transit. No significant findings were observed in other abdominal organs or the peritoneal cavity.

**Figure 1:**
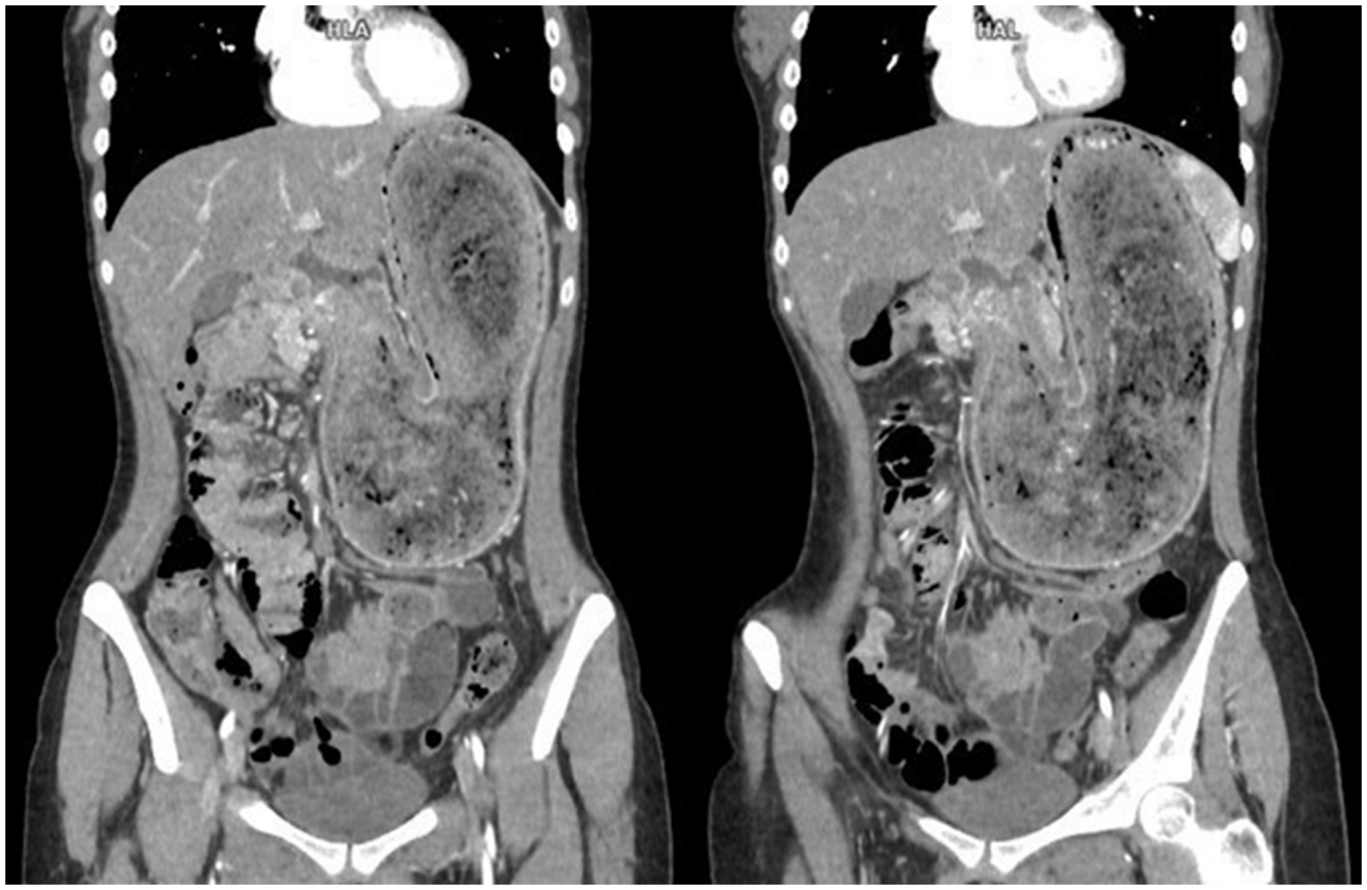
Computerized tomography (CT) images in the coronal plane after the intravenous contrast agent administration in the arterial phase. The images show an extremely dilated stomach filled with a clearly demarcated, compact, bizarre, inhomogeneous mass with interposed air inclusions and no postcontrast enhancement.

Since the indirect ultrasound findings indicated bowel obstruction, the radiologist suggested an abdominal computerized tomography (CT) scan with intravenous contrast. The CT scan revealed a significantly distended stomach, displacing surrounding structures, with a clearly demarcated intraluminal mass extending from the gastric cardia through the pylorus into the duodenum and proximal jejunal loops (Figure 1), with no signs of bowel wall ischemia. The mass was described as a banded, compact, bizarre, inhomogeneous formation with interposed air inclusions and no postcontrast enhancement. A diagnosis of Rapunzel syndrome was strongly suggested.

**Figure 2:**
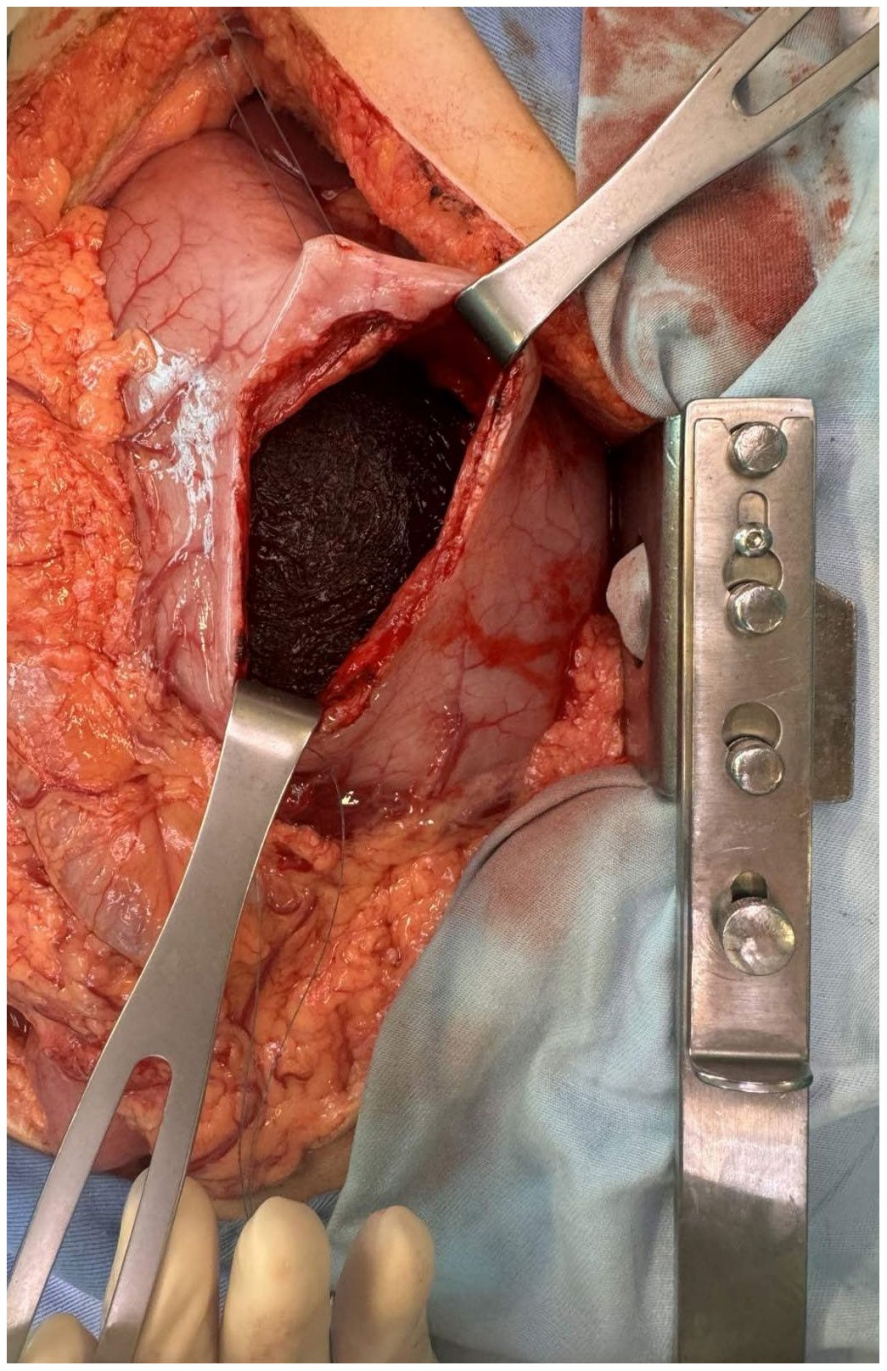
Intraoperative image during gastrotomy showing a large brown intraluminal mass of entangled hair, representing a trichobezoar.

When questioned on multiple occasions, the patient denied ingesting hair or any other unusual materials.

Upon admission, intensive conservative therapy was initiated, including fluid resuscitation, acid–base and electrolyte status correction, and antibiotic therapy.

Given the clinical and imaging findings of the described trichobezoar, surgical treatment was indicated, as it was decided that conservative or endoscopic treatment would not be appropriate or sufficient due to the size and extent of the mass.

**Figure 3:**
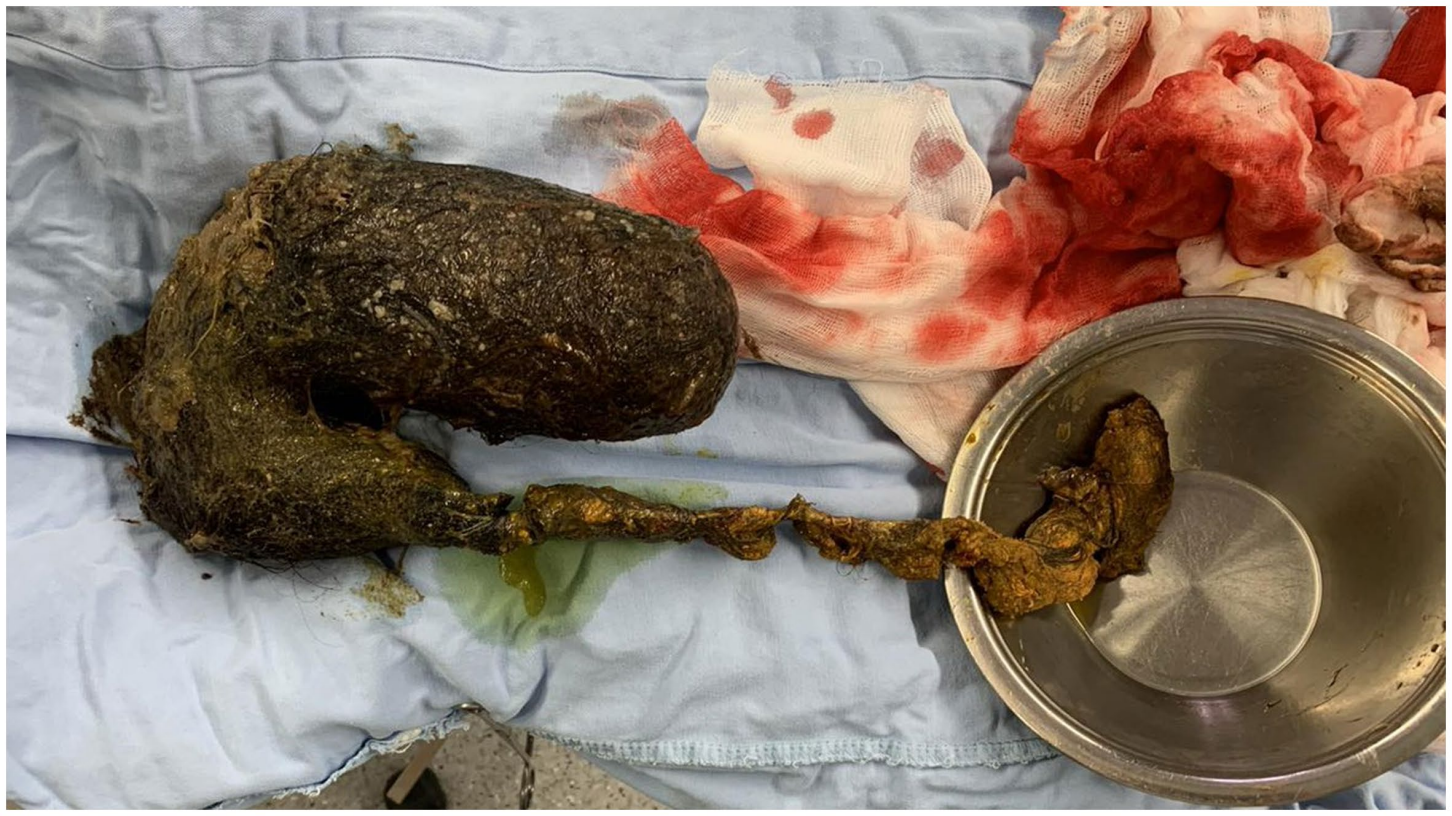
Completely evacuated trichobezoar with a long tail, which was intraoperatively found to extend to the proximal jejunal loops.

A midline laparotomy with exploration was performed. The intraoperative findings revealed an enlarged, distended stomach filled with firm, solid content. Palpation and examination of the bowel revealed that the mass extended through the pylorus into the duodenum and the proximal 20 cm of the jejunum. Therefore, a gastrotomy with a prepyloric incision was performed to enable complete removal of the mass while preserving the pylorus. In addition, intraoperative endoscopy was performed to confirm the absence of any residual trichobezoar. During gastrotomy, a trichobezoar mass resembling a cast of the stomach was evident (Figure 2). Foreign body evacuation was followed by gastric lavage, which revealed intact mucosa. Intraoperative endoscopy showed no residual foreign bodies. The completely removed trichobezoar, including its long tail, is shown in Figure 3. Finally, the gastric wall was sutured in two continuous layers. The patient tolerated the treatment well and had no postoperative complications. A psychiatric examination was conducted following recovery, during which the patient once again denied trichophagia, stating that she had “no clue how the hair could have ended up in her bowel.” No underlying psychiatric conditions or stressors from childhood or later life were identified. A thorough psychiatric examination upon discharge from the hospital was advised. The patient recovered and was discharged on the 7th postoperative day. Ambulatory follow-up assessments showed no complications, resulting in a full recovery.

## Discussion

Trichobezoar is a rare condition characterized by the formation of a hairball in the proximal GI tract. It occurs when ingested hair resists both digestion and peristalsis because of its smooth, slippery surface, accumulating between the mucosal folds of the stomach. With further impaction, as hair particles, mucus, food, and air become trapped, an indigestible mass forms and continuously grows. In most cases, it remains confined to the stomach, usually assuming the shape of a single solid mass (9, 10).

It has been reported that the first mention of a bezoar in a human dates back to a 1779 autopsy of a patient with gastric perforation and subsequent lethal peritonitis (10). Rapunzel syndrome was first described by Vaughan et al. in 1968 (11), who named it after the long-haired protagonist of the famous fairy tale. In our patient, the trichobezoar extended from the stomach through the pylorus to the proximal jejunum, forming a long tail and resulting in a genuine case of Rapunzel syndrome.

There have been reported cases of trichobezoar formation involving materials other than human hair, such as artificial doll hair, cotton, and various fabric fibers (5, 10).

The literature suggests that the formation of bezoars is rare in healthy individuals, typically occurring in patients with a history of gastric surgery or vagotomy, which leads to impaired gastric emptying, or in conditions that cause gastric dysmotility and atony (3). However, this is not the case with trichobezoars as they most frequently occur in young and adolescent women with no history of gastric surgery or dysmotility. Although the literature links the occurrence of trichobezoars to various psychiatric conditions such as emotional problems, family discord, a history of neglect, child abuse, intellectual disability, depression, anxiety, or sudden emotional events in the family (5, 7, 10, 12), some studies propose that only trichotillomania and trichophagia are compulsive behaviors without clear underlying psychiatric conditions mentioned above (5, 8, 13), as was the case with our patient.

This study lacks a detailed psychiatric evaluation of the patient, which could have provided valuable insights into any potential underlying conditions contributing to the compulsive behaviors of trichotillomania and trichophagia. It is estimated that only one-third of patients with trichobezoars experience trichophagia (14). A trichobezoar that requires surgery forms in only approximately 1% of individuals with severe trichotillomania (15). Nevertheless, it is often difficult to determine the cause of trichobezoars in patients with no psychiatric history. However, in the majority of cases, there are varying levels of behavioral disturbances. Therefore, further psychiatric evaluation is essential to exclude more serious conditions, along with psychiatric follow-up to prevent recurrence (16).

The clinical presentation can vary from being asymptomatic or experiencing non-specific intermittent upper GI symptoms—such as epigastric discomfort and pain, abdominal distention, loss of appetite, and weight loss—to showing signs and symptoms of gastric outlet obstruction—such as nausea, vomiting, and constipation (17). The most common presenting sign is typically a palpable mass in the upper abdomen (18). Similar to our patient’s presentation, these non-specific symptoms could persist for an extended period before a diagnosis is made. Therefore, clinical suspicion and careful examination in the appropriate clinical setting could reveal a history of trichophagia.

These patients may have breath with a putrid odor because of the decomposition and fermentation of fats (10), which, along with other information and clinical history, can raise suspicion for a gastric bezoar.

Complications may arise due to the large size of the intragastric mass, which leads to high intraluminal pressure and subsequent erosions, ulceration, or gastric emphysema. The pressure from the mass can result in biliary obstruction or acute pancreatitis (10, 18, 19), consequently leading to additional symptoms such as upper GI bleeding or even jaundice. In rare cases, it can lead to complete gastric obstruction with perforation and the development of an acute abdomen (18, 20).

Given that patients most commonly present with the mentioned signs and symptoms of upper GI conditions, the initial diagnostic procedures include a plain abdominal radiograph and an abdominal ultrasound, followed by an Esophagoastroduodenoscopy (EGDS), which is considered the gold standard for diagnosis (14, 16, 17). The former two modalities are somewhat unreliable with unsatisfactory sensitivity (14, 21, 22).

A CT scan with intravenous contrast medium is the most useful and precise radiological modality for diagnosing trichobezoars, with its results highly coinciding with surgical findings (23). It not only confirms the presence of an intraluminal mass but can also assess its composition, exact size, propagation, and level of obstruction, as well as the condition of the bowel wall, signs of bowel distress, or even perforation (21). The specificity of our case report lies in the precise preoperative radiological diagnosis of the extension of the trichobezoar into the proximal jejunum. The CT evidence of the trichobezoar’s presence in the small bowel was of great importance for planning the optimal therapeutic approach, including the choice of optimal gastrotomy localization and the use of intraoperative endoscopy during the trichobezoar extraction. This approach ultimately enabled the preservation of the pylorus while completely evacuating the foreign body.

Typical postcontrast CT findings include a well-defined, oval intraluminal mass of heterogeneous density, sometimes described as concentric band layers in the form of an onion bulb, with no contrast enhancement and air bubbles retained within the interstices (21, 24). This description aligns well with the findings presented in our case, which showed no complications or signs of bowel wall abnormalities. Given the satisfactory postoperative follow-up, the CT findings corresponded well not only with the intraoperative findings but also with the condition of the bowel itself.

Different therapeutic options are available for removing gastric bezoars, including both operative and non-operative methods. These methods include the administration of prokinetic agents, lytic enzymes, and Coca-Cola®-lysis for chemical dissolution. Although non-invasive, these options carry a considerable risk of developing gastric ulcers (25, 26). The most effective non-invasive option is endoscopy with fragmentation and aspiration (25), but it is often unsuccessful in severe and extensive cases, such as Rapunzel syndrome, and is not without complications, such as small bowel obstruction or even perforation (27, 28). Surgical options include laparoscopy and conventional laparotomy with gastrotomy, which is still considered the treatment of choice, especially for large trichobezoars or their associated complications (26, 29, 30). Some studies have reported cases of combined laparoscopy and endoscopy cooperative surgery (LECS) for the removal of gastric bezoar, showing promising results (31–33). A midline laparotomy with prepyloric gastrotomy and foreign body evacuation was considered the best choice for our patient because of the size of the trichobezoar, and it proved to be both effective and safe as the postoperative course progressed without complications.

## Conclusion

Although rare, trichobezoars should be considered in young women who present with prolonged upper GI symptoms or even gastric outlet obstruction, especially if an underlying psychiatric disorder is present, as they can lead to some serious gastric and abdominal complications. Timely treatment, whether non-surgical or surgical, can relieve the symptoms and prevent complications. A psychiatric examination and follow-up are necessary to diagnose any underlying disorders and prevent recurrence.

## Data Availability

The raw data supporting the conclusions of this article will be made available by the authors, without undue reservation.
