# Supplementary figures and images for: Case Report on Rapunzel syndrome: a large gastric trichobezoar extending to the proximal jejunum in a young adult female
Source: Front Med (Lausanne). 2025 Mar 19;12:1504822. doi: 10.3389/fmed.2025.1504822 (PMC11962009; doi:10.3389/fmed.2025.1504822)

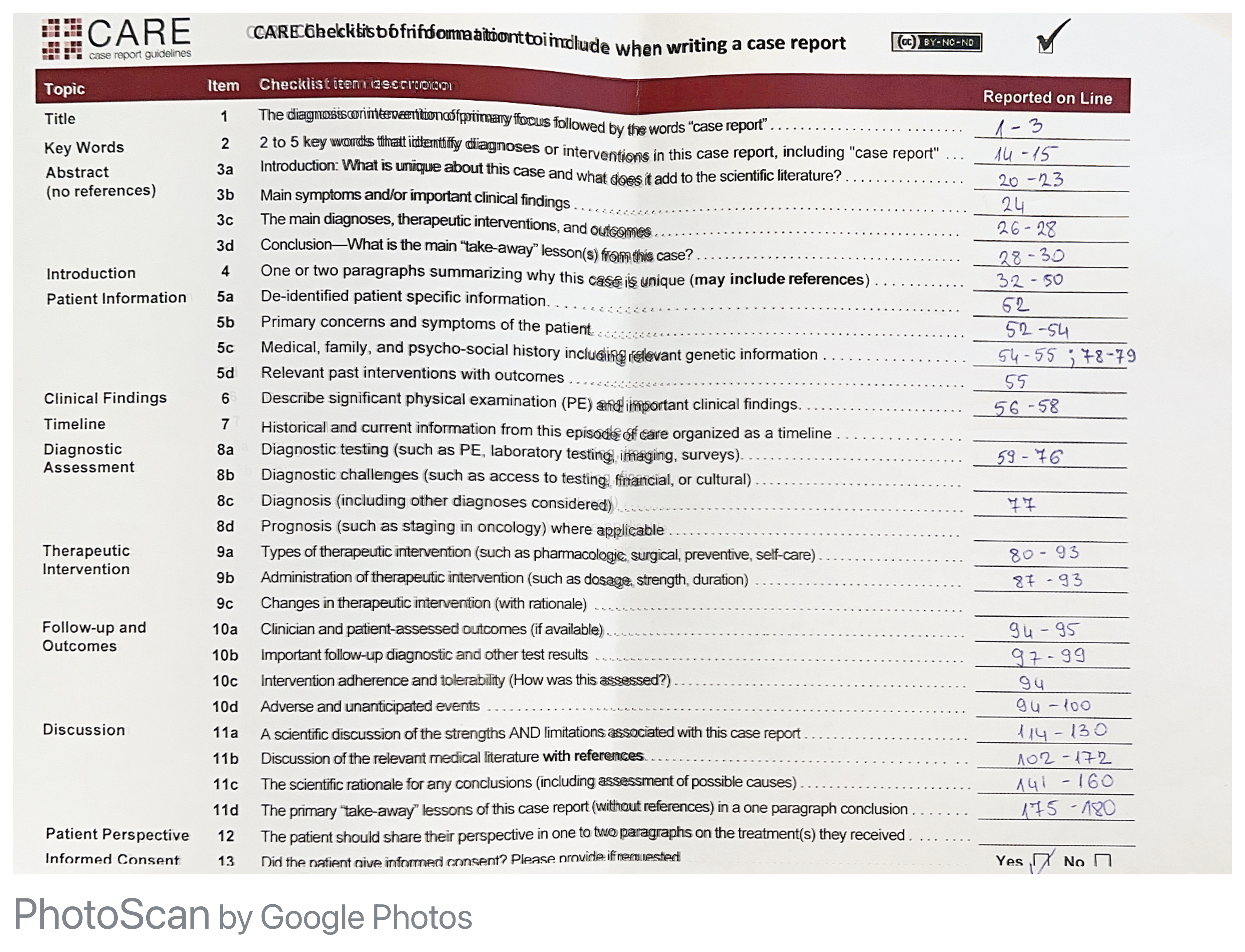

Supplement: Supplementary file 1 [file Image_1.JPEG]
